# Autoimmune polyglandular syndrome type 1 with compound heterozygous *AIRE* gene pathogenic variants and stage 1 type 1 diabetes mellitus: case report and literature review of Chinese population

**DOI:** 10.3389/fimmu.2026.1781304

**Published:** 2026-04-01

**Authors:** Siruo Liu, Conghui Cao, Xiaoli Wang

**Affiliations:** 1 First Department of Infectious Diseases, The First Hospital of China Medical University, Shenyang, China; 2 Department of Endocrinology and Metabolism, NHC Key Laboratory of Diagnosis and Treatment of Thyroid Diseases, Institute of Endocrinology, The First Hospital of China Medical University, Shenyang, China

**Keywords:** aire gene, autoimmune polyendocrine syndrome type 1, hypoparathyroidism, type 1 diabetes mellitus, vitiligo

## Abstract

**Background:**

Autoimmune polyendocrine syndrome type 1 (APS-1) is a rare monogenic autoimmune disorder caused by pathogenic variants in the AIRE gene, characterized by impaired central immune tolerance and multi-organ autoimmune damage. While relatively common in genetically isolated populations, genetically confirmed APS-1 cases remain exceptionally rare in Chinese individuals. To date, population-specific genotypic and phenotypic features of APS-1 in China have not been systematically summarized.

**Case presentation:**

We report a 31-year-old female patient who presented with hypocalcemic convulsions as the initial symptom, accompanied by a 20-year history of vitiligo and mild anemia, newly developed chronic diarrhea and positive islet autoimmunity. Laboratory examinations confirmed hypoparathyroidism and stage 1 type 1 diabetes mellitus (T1DM) with significantly elevated islet autoantibodies but normal islet function. Genetic analysis identified novel compound heterozygous pathogenic variants in the AIRE gene: a missense variant c.977C>T (p.Pro326Leu) inherited from her mother and a 1.6 kb deletion spanning exons 2–4 with an untraceable origin due to the lack of paternal specimen, both classified as pathogenic according to ACMG guidelines.

**Conclusion:**

We performed a systematic narrative review integrating 24 previously reported genetically confirmed Chinese APS-1 cases, forming a combined cohort of 25 cases for comprehensive analysis. This study identified the deletion of *AIRE* gene exons 2–4 as a recurrent pathogenic variant observed in Chinese APS-1 patients, and revealed distinct phenotypic patterns of Chinese patients including a male-to-female ratio of 2:1, a low incidence of the classic triad (44%) and a 16% prevalence of pancreatic autoimmunity. As the first genetically confirmed Chinese case of APS-1 complicated with stage 1 T1DM, this report fills the gap in early pancreatic autoimmunity phenotypic data for Chinese APS-1 patients and enriches the disease’s clinical and genetic spectrum. Clinicians should suspect APS-1 and prioritize early *AIRE* gene testing in young patients with non-surgical hypoparathyroidism and concurrent autoimmune manifestations to prevent misdiagnosis or delayed diagnosis.

## 1 Introduction

Autoimmune polyglandular syndrome type 1 (APS-1) is a rare hereditary autoimmune disorder characterized by immune tolerance loss and organ-specific autoimmune attacks, with classic triad including chronic mucocutaneous candidiasis (CMC), hypoparathyroidism (HP), and autoimmune adrenal insufficiency (AD) (1). Some patients present with multi-organ involvement and complications such as T1DM, autoimmune thyroid disease and vitiligo (1, 2).

APS-1 is mainly caused by variants of the *AIRE* gene (chr21q22.3), whose dysfunction impairs central immune tolerance and triggers autoreactive T-cell-mediated target organ damage (1). Over 200 AIRE gene variants with heterogeneous pathogenicity and phenotypes have been identified (HGMD), and the prevalence of APS-1 is higher in genetically isolated populations (3).

Genetically confirmed APS-1 cases are scarce in the Chinese population, with no systematic summary of genotypic/phenotypic characteristics. To date, only 24 genetically confirmed Chinese APS-1 cases have been reported, and this study adds 1 new case (4–14). By reporting this APS-1 case with compound heterozygous *AIRE* gene variants complicated with stage 1 T1DM and conducting a systematic literature review, this study aims to supplement domestic data, clarify recurrent genetic variants and distinct phenotypic patterns of Chinese APS-1, and provide references for clinical diagnosis, treatment and genetic counseling.

## 2 Case presentation

### 2.1 Clinical and immunological characteristics

A 31-year-old female was admitted for numbness and convulsions of hands and feet for over 6 months (worsened in 2 months). She developed hand-foot numbness 1 year ago without attention, with aggravated symptoms and occasional convulsions 6 months ago. She was diagnosed with hypoparathyroidism in a local hospital 3 months ago (decreased serum calcium and PTH), with mild symptom relief after oral calcium, calcitriol and vitamin D supplements.

The patient had 4-month chronic intermittent watery diarrhea (5–6 times/day, no blood/pus) with ~5 kg weight loss, 20 years of vitiligo and 20 years of mild anemia. Her mother had T2DM, and father was healthy.

Physical examination: Height 166 cm; weight 46 kg; BMI 16.7 kg/m²; scattered skin depigmented macules; no other abnormalities.

The key results of laboratory examinations are summarized in **Table 1**, with abnormal indicators as follows: Glucose metabolism and islet function: Fasting and oral glucose tolerance test (OGTT) blood glucose, insulin and C-peptide levels were all within the normal range, indicating normal basal and reserve function of pancreatic islet β-cells; however, islet-related autoantibodies [glutamic acid decarboxylase antibody (GAD): 203.40 IU/mL, islet cell antibody (ICA): >400.00 IU/mL] were significantly elevated, consistent with the diagnosis of stage 1 T1DM. Parathyroid function: Decreased serum calcium (1.86 mmol/L), increased phosphorus (1.63 mmol/L) and reduced PTH (8.74 pg/mL) levels confirmed the diagnosis of hypoparathyroidism. Autoantibody profile: Anti-Ro-52 antibody was 3+ positive, antinuclear antibody (ANA) was weakly positive at a titer of 1:100, and other autoantibodies including anti-double-stranded DNA antibody (DsDNA) were negative. This low-titer ANA positivity was considered clinically insignificant with no evidence of systemic autoimmune disease. Other endocrine functions: Thyroid function was normal with only a mild elevation of thyroid peroxidase antibody (TPOAb); adrenal function and gonadal function showed no obvious abnormalities. The routine blood test: Hemoglobin level of 106 g/L, indicating mild anemia.

**Table 1:** Laboratory examination results.

Examination category	Test item	At diagnosis		Follow up (3 months)	Reference range
Glucose Metabolism Assessment	OGTT	0 min	120 min	0 min	
	PG (mmol/L)	4.87	6.2	4.65	4.4-6.0
	INS (mIU/L)	2.75	16.49	5.02	4.02-23.46
	CP (ng/dl)	0.75	3.45	1.37	0.929-3.73
	HbA1c (%)	5.4		5.5	4.0-6.0
Islet Autoantibodies	IAA (IU/mL)	4.29		4.17	0.41-20
	GAD (IU/mL)	203.4		212.5	0-17
	IA-2 (IU/mL)	<2.00		<2.00	0.00-10.00
	ICA (IU/mL)	>400.00		>400.00	0.00-20.00
Routine Blood Test	Hb (g/L)	106		108	115-150
Blood Electrolyte and Parathyroid Function	Ca (mmol/L)	1.86		2.18	2.11-2.62
	P (mmol/L)	1.63		1.63	0.85-1.51
	PTH (pg/mL)	8.74		7.98	15.00-65.00
Thyroid Function and antibodies	FT4 (pmol/L)	12.16		12.87	9.01-19.05
	FT3 (pmol/L)	3.09		3.16	2.43-6.01
	TSH (mIU/L)	1.52		1.49	0.35-4.94
	TPOAb (IU/mL)	8.71		9.21	0.00-5.61
	TGAb (IU/mL)	1.66		1.75	0.00-4.10
	TRAb (IU/L)	0.80		0.80	0.00-1.75
Adrenal Function	ACTH (pg/mL)	11.53		11.48	7.2-63.3
	COR (nmol/L)	442.00		398.00	64.00-327.00
Sex Hormones	LH (mIU/mL)	15.90		–	1.1-11.6
	FSH (mIU/mL)	5.07		–	2.8-11.3
	E2 (pmol/L)	309.7		–	45.40-854.00
Autoantibody Spectrum	ANA	+1:100		–	Negative
	DsDNA	Negative		–	Negative
	U1RNP	Negative		–	Negative
	SM	Negative		–	Negative
	SSA	Negative		–	Negative
	Ro-52	3+		–	Negative
	SSB	Negative		–	Negative
	SCL-70	Negative		–	Negative
	PM-Scl	Negative		–	Negative
	JO-1	Negative		–	Negative
	ANUA	Negative		–	Negative
	AHA	Negative		–	Negative
	P	Negative		–	Negative
	AMA-M2	Negative		–	Negative
	PCNA	Negative		–	Negative
	CENP B	Negative		–	Negative
	LKM-1	Negative		–	Negative
	LC-1	Negative		–	Negative
	SLA/LP	Negative		–	Negative
	PML	Negative		–	Negative
	Sp100	Negative		–	Negative
	Gp210	Negative		–	Negative
	M2-3E	Negative		–	Negative

OGTT, Oral glucose tolerance test; PG, plasma glucose; INS, insulin; CP, C-peptide; HbA1c, glycated hemoglobin A1c; IAA, insulin autoantibody; GAD, glutamic acid decarboxylase antibody; IA-2, insulinoma-associated protein 2 antibody; ICA, islet cell antibody; Hb, hemoglobin; Ca, calcium; P, phosphorus; PTH, parathyroid hormone; FT4, free thyroxine; FT3, free triiodothyronine; TSH, thyroid-stimulating hormone; TPOAb, thyroid peroxidase antibody; TGAb, thyroglobulin antibody; TRAb, thyroid-stimulating hormone receptor antibody; ACTH, adrenocorticotropic hormone; COR, cortisol; LH, luteinizing hormone; FSH, follicle-stimulating hormone; E2, estradiol; ANA, antinuclear antibody; DsDNA, anti-double-stranded DNA antibody; U1RNP, anti-U1 ribonucleoprotein antibody; SM, anti-Sm antibody; SSA, anti-SSA antibody; Ro-52, anti-Ro-52 antibody; SSB, anti-SSB antibody; SCL-70, anti-scleroderma-70 antibody; PM-Scl, anti-PM-Scl antibody; JO-1, anti-JO-1 antibody; ANUA, anti-nucleosome antibody; AHA, anti-histone antibody; P, anti-ribosomal P protein antibody; AMA-M2, anti-mitochondrial antibody M2 subtype; PCNA, anti-proliferating cell nuclear antigen antibody; CENP B, anti-centromere protein B antibody; LKM-1, anti-liver kidney microsome antibody type 1; LC-1, anti-liver cytosol antibody type 1; SLA/LP, anti-soluble liver antigen/liver pancreas antibody; PML, anti-promyelocytic leukemia protein antibody; Sp100, anti-Sp100 nuclear antigen antibody; Gp210, anti-glycoprotein 210 antibody; M2-3E, anti-mitochondrial antibody M2-3E subtype.

The patient had chronic intermittent watery diarrhea for 4 months, tentatively attributed to intestinal mucosal immune dysfunction secondary to *AIRE* gene variant-induced central immune tolerance deficiency, a non-classic manifestation of APS-1. As the diarrhea had alleviated on admission, specific diagnostic assessments for autoimmune enteropathy were not conducted, limiting the clinical characterization of this gastrointestinal finding.

### 2.2 Genetic analysis

**Figure 1:**
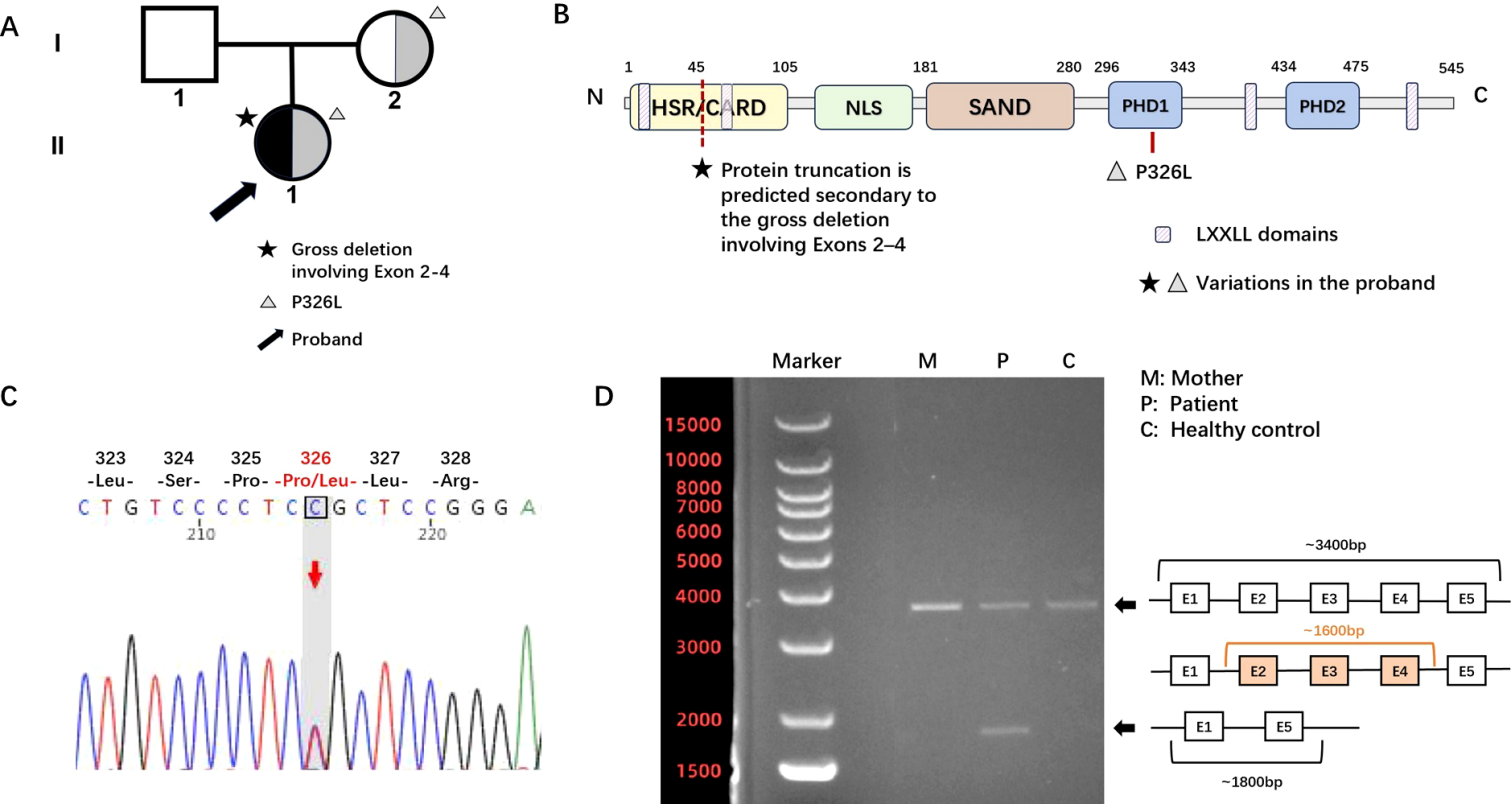
Pedigree chart and genetic analysis. **(A)** Pedigree chart. The proband (arrow-indicated) carries compound heterozygous pathogenic variants of the *AIRE* gene. The pentagram (★) denotes the gross deletion involving exons 2-4, and the triangle (△) denotes the missense variant p.Pro326Leu (P326L). Genetic analysis confirmed that the p.Pro326Leu variant was inherited from her mother. No specimen of the father was available for detection, so the origin of the gross deletion variant could not be traced. **(B)** Schematic diagram of the *AIRE* gene and variant locations. The *AIRE* gene encodes a protein with multiple functional domains, including the Homogeneously Staining Region/Caspase Recruitment Domain (HSR/CARD, amino acids 1–105), a nuclear localization signal (NLS, amino acids 100–189), the SAND domain [SAND: Sp100, AIRE, NucP41/75, and DEAF-1 domain, amino acids 181–280], two plant homeodomain (PHD) finger-type zinc fingers (PHD, amino acids 296–343 and 434–475), and four LXXLL domains (amino acids 7–11, 63–67, 414–418, and 516–520) that function as coactivators of nuclear receptors. The missense variant p.Pro326Leu is located in the first PHD domain, while the gross deletion variant involves exons 2–4 of the *AIRE* gene. **(C)** Sanger sequencing electrophoretogram verifying the p.Pro326Leu variant. The electrophoretogram confirms the presence of the c.977C>T (p.Pro326Leu) missense variant in the patient, which is consistent with the heterozygous variant pattern; the mother also carries this variant, while the wild-type sequence is shown as a reference. **(D)** PCR amplification results of the *AIRE* gene fragment. PCR amplification was performed on DNA fragments from the patient, her mother, and a healthy control using specific primers (forward: CTGGCCGCCTTCCTTCTAAAGCA; reverse: ACCCTCCTCCTCCAAAGTCAGCA). The amplification range was designed to span exon 1 to intron 5, and typical long-fragment PCR conditions were applied. The results showed that the patient had a deletion of approximately 1600 bp in this fragment, while the mother and healthy control exhibited normal amplification products without deletion.

Whole-exome sequencing (WES) and copy number variation (CNV) analysis were performed on the patient and her mother (no paternal specimen), without additional techniques such as MLPA or CMA. The results revealed that the patient carried compound heterozygous pathogenic variants in the *AIRE* gene, including one missense variant [c.977C>T (p.Pro326Leu)] and one large-fragment deletion variant. Sanger sequencing combined with polymerase chain reaction (PCR) amplification was further conducted for variant verification and inheritance pattern analysis (**Figure 1**).

The missense variant c.977C>T (p.Pro326Leu) (exon 8, NM_000383.4) was maternally inherited, with an extremely low allele frequency (0.00001750, gnomAD). This variant has been reported as homozygous/compound heterozygous in multiple patients with APS (15, 16), and another substitution at the same locus (p.Pro326Gln) was also reported in such patients (17). This missense variant was classified as pathogenic (PS4, PM2, PM3, PM5, PP3, PP4, PP5) according to the ACMG variant interpretation guidelines.

Meanwhile, WES data analysis indicated a copy number deletion signal of approximately 1.6 Kb in the genomic DNA chr21q22.3 region of the patient. Family NGS analysis and PCR amplification confirmed that the patient’s mother did not carry this deletion variant, and the origin of this variant could not be traced due to the lack of the father’s specimen. The copy number deletion region mainly involves exons 2–4 of the *AIRE* gene and adjacent introns, which constitutes a frameshift variant caused by a non-triple base deletion. This deletion variant may lead to the loss of normal AIRE protein function through nonsense-mediated mRNA decay (NMD) or premature termination of the encoded amino acid sequence. This deletion variant was also classified as pathogenic (PVS1, PM2, PM3, PP4) based on the ACMG guidelines.

Based on the clinical manifestations and genetic testing results, APS-1 was diagnosed.

### 2.3 Treatment and follow-up

The patient received an individualized treatment regimen with regular follow-up for dynamic adjustment. For hypoparathyroidism, oral calcium, calcitriol and vitamin D supplements were continued, with serum calcium normalized to 2.18 mmol/L at the 3-month follow-up and no numbness or convulsion recurrence. The anti-CD3 monoclonal antibody teplizumab was recommended for stage 1 T1DM but declined by the patient due to cost concerns. Probiotics were administered for symptomatic relief of diarrhea, and the patient was referred to a dermatologist for vitiligo management. Regular monitoring of thyroid function, islet function and autoantibodies was advised; a 3-month reexamination showed stable blood glucose, preserved islet function and no worsening of autoimmune indicators. The patient was instructed to maintain a regular lifestyle and undergo routine reexaminations of serum calcium, phosphorus, PTH, blood routine, thyroid and islet function to monitor disease progression and adjust medications timely.

## 3 Discussion

### 3.1 Literature search and selection methods

A systematic narrative literature review was performed to summarize the genetically confirmed cases of APS-1 in the Chinese population. Literature retrieval was conducted across major Chinese (CNKI, Wanfang, and VIP) and English (PubMed, EMBASE, and Web of Science) databases, covering all records from database inception to December 2025. The search terms were “Autoimmune polyendocrine syndrome type 1, APS-1, *AIRE* gene, Chinese”. Inclusion criteria: ① Definite diagnosis of APS-1; ② Confirmation of *AIRE* gene variants by Sanger sequencing, whole-exome sequencing, MLPA or other methods; ③ Study subjects were the Chinese population; ④ Complete clinical phenotypic and genetic variant data were provided. Exclusion criteria: ① Cases with only clinical diagnosis without genetic testing; ② Cases with unclear genetic testing results; ③ Cases repeatedly published in different journals (only the first published one or the one with the most complete data was included).

According to the above search and selection criteria, 11 studies involving 24 genetically confirmed APS-1 cases in the Chinese population were finally included (4–14). Combined with the 1 case reported in this study, a total of 25 cases were included in the subsequent analysis, which is the most comprehensive systematic summary of APS-1 cases in the Chinese population to date (**Table 2**).

**Table 2:** Clinical spectrum and genetic variants of genetically confirmed APS-1 cases in the Chinese population.

Case	Age at last visit (year)	Age at onset (year)	Sex	Clinical manifestation				*AIRE* variations	Heterozygosity	Reference
				HP	CMC	AD	Other			
1	18	0.8	Female	+	+	–	HT, ED, A, RP	c.38T>C, p.L13P	Het	(4)
2	30	12	Male	+	+	+	A	c.47C>G, p.T16R; c.1631–2 A>T	Com Het	
3	14	4	Female	+	+	+	ED	c.55G>A, p.A19T; c.769C>T, p.R257X	Com Het	
4	19	2	male	+	–	+	ED, IM, RTA	c.206A>C, p.Q69P	Hom	
5	28	5	Female	+	+	+	HT, ED, A, HO	c.269A>G, p.Y90C	Hom	
6#	24	18	Male	–	–	+	T1DM, K, PA	c.463G>A, p.G155S	Hom	
7#	21	1	Female	+	+	–	JE	c.463G>A, p.G155S	Hom	
8	23	15.6	Male	+	+	+	HT, A	c.483_484insC, p.K161fs	Hom	
9	27	12	Female	+	+	+	HT	c.483_484insC, p.K161fs	Hom	
10	15	12.5	Female	+	+	–	ED, IM	c.622G>T, p.G208W	Het	
11	32	6.3	Female	+	–	–	HG, IM, HO	c.623G>T, p.G208V	Het	
12	10	5	Male	+	+	–	ED	c.737delC, p.A246fs; c.922C>T, p.L308F	Com Het	
13	9	8.4	Male	+	+	+	–	Entire gene; IVS11 + 1G>A	Com Het	
14	42	42	Male	+	+	+	HG	c.415C>T, p.R139X	Hom	(5)
15	15	15	Male	+	+	+	–	c.47C>G, p.T16R	Hom	(6)
16	20	9	Male	–	+	+	HT, A, CD	c.239T>G, p.V80G; Exon1–2 del	Com Het	(7)
17	31	7	Female	+	–	–	HG, IM, PRCA, LGLL	c.371C>T, p.P124L; c.623G>T, p.G208V	Com Het	(8)
18	2	2	Male	–	+	–	RP	c.769C>T, p.R257X	Hom	(9)
19	15	12	Female	+	+	+	–	c.74C>G, p.A25G	Hom	(10)
20	34	10	Male	+	+	+	HT	c.179A>G, p.H60R; c.463 + 2T>C	Com Het	(11)
21	12	3	Male	–	+	+	PA	c.44G>A, p.R15H; c.1036C>T, p.Q346X	Com Het	(12)
22	17	7	Male	+	+	+	PA	c.38T>C, p.L13P; c1400 + 2T>C	Com Het	(12)
23	16	12	Male	+	+	+	T1DM	Exon2–4 del	Hom	(13)
24	21	17	Male	–	+	–	T1DM	Exon2–4 del	Hom	(14)
25	31	11	Female	+	–	–	V, IM, T1DM stage 1	c.977C>T, p.Pro326Leu; Exon2–4 del	Com Het	This study

HP, hypoparathyroidism; CMC, chronic mucocutaneous candidiasis; AD, Addison’s disease; HT, hypothyroidism; HG, hypergonadotropic hypogonadism; T1DM, type 1 diabetes mellitus; ED, ectodermal dysplasias, including enamel dysplasia and nail dystrophy; A, alopecia; K, keratitis; RP, retinitis pigmentosa; IM, intestinal malabsorption; HO, hematopathy; RTA, renal tubular acidosis; PA, pernicious anemia; CD, central diabetes insipidus; JE, Japanese encephalitis; PRCA, pure red cell aplasia; LGLL, large granular lymphocytic leukemia; V, vitiligo; Het, heterozygous; Hom, homozygous; Com Het, compound heterozygous.

### 3.2 Analysis of clinical phenotypic characteristics of Chinese patients with APS-1

The patient developed vitiligo at the age of 11 years and was diagnosed with APS-1 at the age of 31 years due to hypocalcemic convulsions, with chronic diarrhea as an important diagnostic clue. This course is consistent with typical APS-1 features: intermittent symptom onset for years, easily ignored initial symptoms, and delayed diagnosis until adulthood.

A special phenomenon was found in this study’s literature search: the same case was repeatedly reported in different journals. For example, Case 18 with homozygous p.Arg257Ter variant was simultaneously reported in three specialist journals (9, 18, 19), and a similar situation was observed in Case 17 (8, 20). APS-1 patients with multiple atypical manifestations are often reported as rare cases by different specialties due to different visiting departments. This phenomenon not only reflects the widespread development of genetic testing for rare diseases in China but also highlights the urgent need to strengthen multidisciplinary communication and collaboration, which is crucial for the comprehensive management of APS-1 patients.

Systematic analysis of phenotypic characteristics in 25 Chinese APS-1 cases (including the present case) revealed distinct clinical features from international reports (1, 21, 22): a male-to-female ratio of 2:1, with 44% (11/25) of patients presenting with the complete classic triad, 32% (8/25) with two components, and 24% (6/25) with only one component; hypoparathyroidism (HP) and chronic mucocutaneous candidiasis (CMC) were the most prevalent components (80% each), while autoimmune adrenal insufficiency (AD) was detected in 64% (16/25) of cases. These findings suggest potential ethnic differences in the clinical phenotype of APS-1.

The present case presented with HP, vitiligo, mild anemia, chronic diarrhea and positive islet autoantibodies without CMC or AD, which is a typical non-classic phenotype and enriches the non-classic phenotypic spectrum of APS-1 in the Chinese population. This case suggests that clinicians should suspect APS-1 in young patients with non-surgical HP combined with other autoimmune manifestations even without the classic triad, and perform *AIRE* gene testing in a timely manner to avoid missed diagnosis and misdiagnosis.

Analysis of atypical autoimmune manifestations in the 25 cases showed that the incidence of gastrointestinal manifestations (diarrhea/intestinal malabsorption) was 20% (5/25), skin manifestations (vitiligo/alopecia) 24% (6/25), and pancreatic autoimmunity/T1DM 16% (4/25). This indicates that the gastrointestinal tract, skin and pancreas are the most common atypically involved organs in Chinese APS-1 patients, and comprehensive evaluation of these organs should be conducted in clinical diagnosis and treatment to avoid missing potential autoimmune damage.

### 3.3 Genetic variant characteristics and population specificity

Analysis of *AIRE* gene variant characteristics in Chinese APS-1 patients reveals several notable findings: the Arg257Ter variant, a common variant in many ethnic groups, was detected in only two Chinese cases (one homozygous), suggesting that this variant is not a high-frequency variant in the Chinese population. The *AIRE* gene exons 2–4 deletion variant was found in three cases in our cohort (3/25), including the present compound heterozygous case and two homozygous cases; this variant is a notable recurrent genetic finding in Chinese APS-1 patients and, to our knowledge, has not been reported in non-Chinese cohorts. Three cases carried only a single heterozygous variant, suggesting possible undetected intronic or large fragment deletions. Comprehensive *AIRE* testing may require more sensitive methods such as MLPA or CNV sequencing.

### 3.4 Interpretation of the immunological mechanism

The core pathogenesis of APS-1 is central immune tolerance deficiency caused by *AIRE* gene variants. The AIRE protein is highly expressed in thymic medullary epithelial cells, and its main function is to mediate the thymic presentation of peripheral tissue antigens, enabling immature T cells to contact peripheral tissue antigens in the thymus, achieving clonal deletion of autoreactive T cells and maintaining central immune tolerance. *AIRE* gene pathogenic variants lead to protein function loss, ineffective peripheral antigen presentation, autoreactive T cell escape, and subsequent multi-organ autoimmune damage (1).

High titers of islet autoantibodies (GAD/ICA) and positive anti-Ro52 antibody were detected in this case, and this autoantibody profile is a direct peripheral manifestation of *AIRE*-mediated central immune tolerance deficiency: due to *AIRE* dysfunction, the thymus cannot effectively present islet antigens and Ro52 antigens, leading to the escape of autoreactive T cells against the above antigens, which in turn activate B cells to produce specific autoantibodies. At the same time, autoreactive T cells directly infiltrate target organs, causing autoimmune damage to the parathyroid gland, intestinal tract, skin and other organs, manifested as clinical symptoms such as HP, autoimmune enteropathy and vitiligo.

IFN-α/ω antibody, a highly specific AIRE-dependent autoantibody for APS-1 diagnosis (positive rate >90%) (1), was not detected due to clinical test limitations, though it is presumed positive based on the patient’s clinical and genetic features. Routine detection of this antibody is recommended in clinical practice to improve the diagnostic specificity of APS-1 in China.

### 3.5 Pancreatic autoimmunity and stage 1 type 1 diabetes mellitus

Islet-related autoantibodies (GAD/ICA) were significantly elevated in this case, while blood glucose and islet function were normal, consistent with the diagnosis of stage 1 T1DM. This is the first reported case of APS-1 complicated with stage 1 T1DM in the Chinese population, filling the gap in early phenotypic data of APS-1 complicated with pancreatic autoimmunity in China.

Wilson et al. recently reported that teplizumab exerts islet protective effects in APS-1 patients with stage 2 T1DM (including reversing one patient to stage 1) with mild adverse reactions, providing evidence for early immunotherapeutic intervention of pancreatic autoimmunity in APS-1 (23). This study confirms that even in the context of central immune tolerance deficiency in APS-1, teplizumab can still exert islet protective effects by regulating T cell phenotype and tissue homing ability, providing direct evidence-based support for the intervention suggestions in this case. This emerging option was informed to the patient, with standardized follow-up established for timely intervention if islet function deteriorates.

### 3.6 Clinical significance of positive anti-Ro52 antibody

The patient had 3+ positive anti-Ro52 antibody, a common autoantibody associated with Sjögren’s syndrome, SLE and autoimmune thyroid disease, but no proven correlation with APS-1. Multiple cohort studies have not reported anti-Ro52 antibodies in APS-1 patients or their association with disease pathogenesis (21, 22).

Anti-Ro52 antibody is not a characteristic APS-1 autoantibody, and its positivity is an occasional finding. The patient had no typical connective tissue disease manifestations and other autoantibodies related to connective tissue diseases were negative, suggesting that the positive anti-Ro52 antibody is not evidence of comorbidity with other connective tissue diseases, consistent with APS-1 as a systemic autoimmune disease caused by AIRE gene variants.

For the clinical management of APS-1 patients with positive anti-Ro52 antibodies, although the specific significance of this antibody remains unclear, it is still necessary to strengthen the comprehensive autoimmune evaluation of systemic multi-organs. This is not due to the specific indicative value of the anti-Ro52 antibody itself, but based on the inherent characteristic of APS-1 as an autoimmune disease involving multiple organs. Regardless of whether the anti-Ro52 antibody is positive or not, regular monitoring of potential autoimmune damage to endocrine and non-endocrine organs is the core of APS-1 clinical management.

It should be emphasized that the clinical significance of anti-Ro52 antibody in APS-1 still needs to be verified by large-sample cohort studies. At present, it cannot be used as a routine monitoring indicator for APS-1, nor can its positivity alone be used to judge the degree of autoimmune activation or predict disease progression.

### 3.7 Study limitations

This study has certain limitations. As a single-case report, the conclusions cannot be generalized to all APS-1 patients, but this study systematically summarized 25 genetically confirmed APS-1 cases in the Chinese population, which compensates for the limitation of the single-case report to a certain extent. Due to the limitation of clinical detection conditions, this study did not perform T/B cell immunophenotyping, detection of AIRE-dependent autoantibodies (IFN-α/ω antibody) and regulatory T cell analysis in the patient, which cannot explain the patient’s immunological characteristics more comprehensively, and this is also an important direction for subsequent research. The long-term follow-up data of the patient is insufficient, and the progression of islet function, autoimmune indicators and other complications needs further observation. This is a narrative literature review, and although strict search and selection criteria were formulated, there is still a certain bias. Subsequent systematic reviews and meta-analyses are needed to more comprehensively summarize the clinical and genetic characteristics of APS-1 in the Chinese population.

## 4 Conclusion

This study reports a non-classic APS-1 case with compound heterozygous *AIRE* gene variants (c.977C>T/p.Pro326Leu and exons 2–4 deletion) complicated with stage 1 T1DM. Systematic analysis of 25 genetically confirmed Chinese APS-1 cases identifies the exons 2–4 deletion as a recurrent pathogenic variant in this population and reveals unique phenotypic features, filling the gap in early pancreatic autoimmunity phenotypic data and enriching the clinical and genetic spectrum of Chinese APS-1. By clarifying the genotypic and phenotypic characteristics of Chinese APS-1 and highlighting its diagnostic challenges, this research supplements the domestic APS-1 database, refines the *AIRE* variant spectrum, and provides valuable references for clinical practice and genetic counseling. Clinicians should suspect APS-1 and perform prompt *AIRE* gene testing in young patients with non-surgical hypoparathyroidism and concurrent autoimmune manifestations to avoid misdiagnosis; individualized treatment and standardized long-term follow-up are crucial for favorable outcomes. Further large-sample, long-term follow-up studies combined with basic experiments are needed to explore the pathogenic mechanisms of population-specific variants and genotype-phenotype correlations, facilitating precise APS-1 management.

## Data Availability

The original contributions presented in the study are included in the article/supplementary material, further inquiries can be directed to the corresponding author/s.
